# A Bispecific Inhibitor of the EGFR/ADAM17 Axis Decreases Cell Proliferation and Migration of EGFR-Dependent Cancer Cells

**DOI:** 10.3390/cancers12020411

**Published:** 2020-02-10

**Authors:** Abel Soto-Gamez, Deng Chen, Anke G.E. Nabuurs, Wim J. Quax, Marco Demaria, Ykelien L. Boersma

**Affiliations:** 1 University of Groningen, Groningen Research Institute of Pharmacy, Chemical and Pharmaceutical Biology, 9713 AV Groningen, The Netherlands; a.a.soto.gamez@rug.nl (A.S.-G.); d.chen@rug.nl (D.C.); a.g.e.nabuurs@student.rug.nl (A.G.E.N.); 2 European Institute for the Biology of Aging (ERIBA), University Medical Center Groningen (UMCG), 9713 AV Groningen, The Netherlands; m.demaria@umcg.nl

**Keywords:** EGFR, ADAM17, bispecifics, DARPins

## Abstract

Dysregulated epidermal growth factor receptor (EGFR) is an oncogenic driver of many human cancers, promoting aberrant cell proliferation, migration, and survival. Pharmacological targeting of EGFR is often challenged by acquired mechanisms of resistance. Ligand-dependent mechanisms in EGFR wild-type cells rely on ligand or receptor overexpression, allowing cells to outcompete inhibitors and perpetuate signaling in an autocrine manner. Importantly, EGFR ligands are synthesized as membrane-bound precursors that must be solubilized to enable receptor-ligand interactions. The A disintegrin and metalloproteinase 17 (ADAM17) is considered the main sheddase of several EGFR ligands, and a potential pharmacological target. However, its broad substrate range and ubiquitous expression complicate its therapeutic targeting. Here, we present a novel bispecific fusion protein construct consisting of the inhibitory prodomain of ADAM17 (TPD), fused to an EGFR-targeting designed ankyrin repeat protein (DARPin). TPD is a natural inhibitor of ADAM17, maintaining the protease in a zymogen-like form. Meanwhile, the high affinity anti-EGFR DARPin E01 binds to EGFR and inhibits ligand binding. The resulting fusion protein E01-GS-TPD retained binding ability to both molecular targets EGFR and ADAM17. The large difference in affinity for each target resulted in enrichment of the fusion protein in EGFR-positive cells compared to EGFR-negative cells, suggesting a possible application in autocrine signaling inhibition. Accordingly, E01-GS-TPD decreased migration and proliferation of EGFR-dependent cell lines with no significant increase in apoptotic cell death. Finally, inhibition of proliferation was observed through EGFR ligand-dependent mechanisms as growth inhibition was not observed in EGFR mutant or KRAS mutant cell lines. The use of bispecific proteins targeting the EGFR/ADAM17 axis could be an innovative strategy for the treatment of EGFR-dependent cancers.

## 1. Introduction

Dysregulated of the epidermal growth factor receptor (EGFR) is an oncogenic driver of many human cancers, promoting aberrant cell proliferation, migration, and survival [1]. Consequently, targeting EGFR using small-molecule tyrosine kinase inhibitors (TKIs) or blocking monoclonal antibodies (mAbs) is common in the clinic. However, tumor cells often develop resistance against both approaches. Dysregulated EGFR signaling and acquired drug resistance can occur in a ligand-independent fashion via mutations leading to constitutive activation of the EGFR receptor [2] or that of downstream signaling components such as KRAS [3]. Alternatively, ligand-dependent mechanisms include ligand and receptor overexpression which allows for increased autocrine signaling [4]. 

EGFR ligands are synthesized as membrane-bound immature precursors and their proteolytic cleavage from the cell surface enables solubilization, diffusion, and receptor binding. The shedding of EGFR ligands, thus, constitutes an additional layer of regulation in establishing receptor-ligand interactions [5]. EGFR-ligand shedding is primarily catalyzed by transmembrane proteinases of the A disintegrin and metalloproteinase (ADAM) family, and ADAM17 (also known as tumor necrosis factor (TNF)-alpha converting enzyme (TACE)) represents the major ‘sheddase’ of the EGFR ligands amphiregulin, heregulin, epiregulin, epigen, and transforming growth factor (TGF)-alpha [6]. ADAM17 also shows sheddase activity towards ligand precursors of EGFR family members Her3 and Her4, thus offering an escape route when signaling through EGFR is inhibited [7].

ADAM17 was originally identified for its role in the release of the pro-inflammatory cytokine TNFα and later proposed to be responsible for the processing of dozens of inflammatory mediators including cytokine receptors (TNF receptor, interleukin-6 receptor) and various cell adhesion molecules (reviewed in [8]). ADAM17 is predominantly activated in disease states associated with infection, autoimmunity, cardiovascular diseases, neurodegeneration, and cancer, in particular non-small cell lung carcinoma (NSCLC) and head-and-neck squamous cell carcinoma (HNSCC) [9,10,11]. Furthermore, ADAM17 can be activated by chemotherapy, resulting in increased ligand shedding [12]. Accordingly, ADAM17 inhibition is beneficial across multiple models of inflammation and cancer, and therefore represents an attractive therapeutic target [4,5,6]. Unfortunately, most ADAM17 small molecule inhibitors display poor specificity and the few with high specificity are associated with serious toxicological issues, presumably due to ADAM17’s multiple roles and ubiquitous expression. Recently, a stable recombinant form of the auto-inhibitory prodomain of ADAM17 (TPD, for TACE prodomain) has been described [13]. Recombinant TPD binds to cell-surface ADAM17 via protein-protein interactions and effectively inhibits proteinase activity both in vitro and in vivo. Moreover, TPD’s inhibitory activity was demonstrated to be specific due to poor homology between the prodomains of other ADAMs and matrix metalloproteinases [13]. 

**Figure 1:**
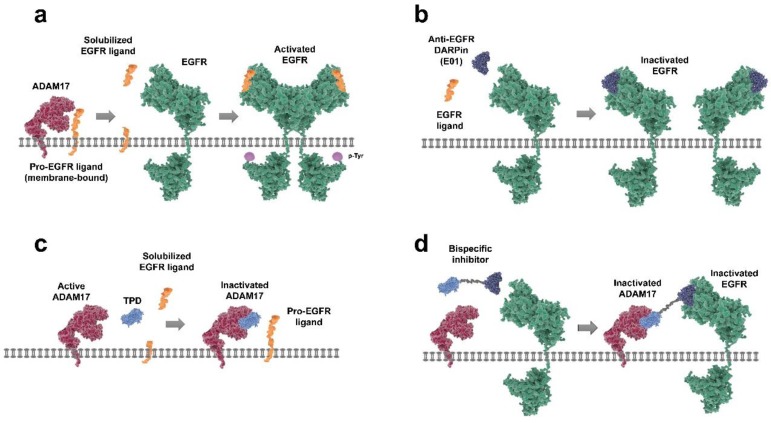
Therapeutic strategies for targeting the epidermal growth factor receptor (EGFR)/A disintegrin and metalloproteinase (ADAM17) axis. (**a**) pro-EGFR ligands are membrane-bound and require proteolytic cleavage by ADAM17 to generate ‘mature’ solubilized ligands that can bind to the EGF receptor. Ligand-bound EGFR can then (homo) dimerize, auto-phosphorylate, and initiate downstream signaling. (**b**) anti-EGFR designed ankyrin repeat protein (DARPin) E01 binds to EGFR in an antagonistic manner, preventing ligand binding and subsequent receptor activation. (**c**) ligand shedding can be prevented by inhibiting ADAM17 activity; for instance, by using recombinant inhibitory prodomain of ADAM17 (TPD) to block the catalytic site of active ADAM17 and preventing release of EGFR ligands. (**d**) the bispecific inhibitor (E01-GS-TPD) combines the activities of DARPin E01 and recombinant TPD by fusing the two modules together through a flexible glycine serine linker.

Dual inhibition of ADAM17 and EGFR may provide enhanced anti-tumor activity by limiting ligand-dependent mechanisms associated with acquired drug resistance. In this study, we aimed to disrupt oncogenic EGFR/ADAM17 signaling using a bispecific fusion protein consisting of the inhibitory prodomain of ADAM17 fused to an anti-EGFR designed ankyrin repeat protein (DARPin) via a flexible glycine serine linker (GS) (Figure 1). DARPins are specialized binding proteins that have been engineered for enhanced biochemical properties compared to antibodies, such as higher stability, solubility, ease of production, and simple customization (reviewed in [14,15]). After production and purification of the recombinant fusion protein E01-GS-TPD, its inhibitory effects were studied across different cell lines with distinct EGFR molecular phenotypes. The activity of both modules in the fusion protein was preserved and the fusion protein effectively inhibited proliferation across EGFR-dependent cell lines.

## 2. Results

### 2.1. Generation of the Fusion Protein E01-GS-TPD

In this study we aimed to disrupt oncogenic EGFR/ADAM17 signaling using a novel bispecific fusion protein consisting of the inhibitory prodomain of ADAM17 (TPD) fused to an anti-EGFR DARPin (E01) via a long flexible Gly-Ser linker (E01-GS-TPD). To this end, we opted for the previously reported DARPin E01, capable of binding domain III of the EGFR ectodomain at low nanomolar affinity in a specific manner, as well as preventing ligand binding and EGFR activation [16,17]. A plasmid encoding the bispecific construct of E01-GS-TPD was generated as previously described [18]. Following production and purification of the recombinant fusion protein (Appendix A), we set out to study its inhibitory effects across different cell lines with distinct EGFR molecular phenotypes. 

### 2.2. E01-GS-TPD Retains Binding Ability to EGFR and ADAM17

**Figure 2:**
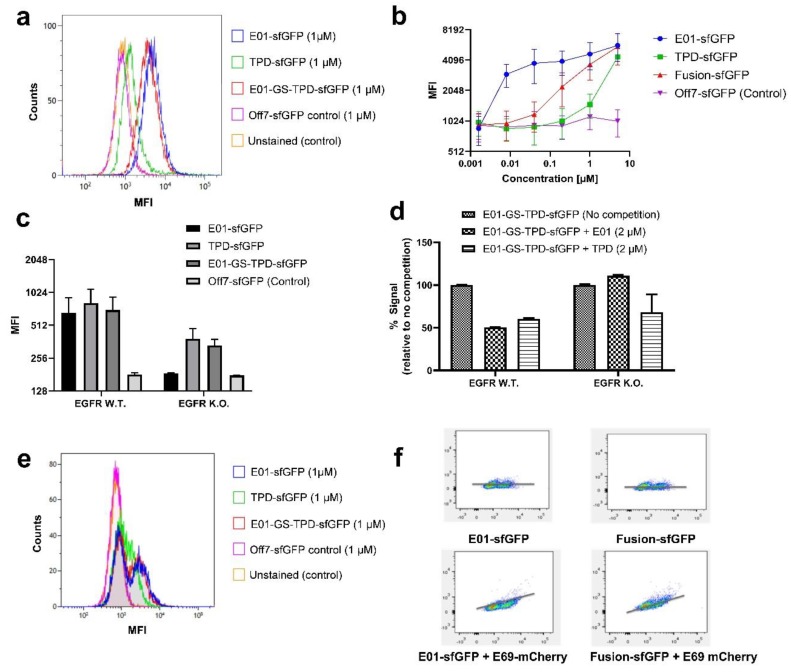
Analytical flow cytometry assays reveal binding of fusion protein E01-GS-TPD. (**a**) overlay histogram of A549 EGFR (+/+) cells treated with the fluorescently tagged recombinant proteins used in this study (mean fluorescence intensities (MFI)). (**b**) binding curves upon incubation of A549 EGFR (+/+) cells with E01-sfGFP, TPD-sfGFP, fusion protein E01-GS-TPD-sfGFP, or negative control DARPin Off7-sfGFP. MFI, and their half-peak coefficient of variation (%CV) are shown for each concentration. (**c**) binding of each protein to A549 EGFR (+/+) or A549 EGFR (−/−) cells. (**d**) competition assays using A549 cells treated with fluorescently labeled fusion protein E01-GS-TPD-sfGFP in competition with unlabeled monomer (E01 or TPD); a decrease in fluorescence was observed in EGFR (+/+) cells; in contrast, no competition from unlabeled E01 for binding to EGFR was observed in EGFR (−/−) cells, while competition from unlabeled TPD remained unaffected. (**e**) overlay histogram of A549 co-cultures of EGFR (+/+) and (−/−) cells (1:1) treated with the fluorescently tagged recombinant proteins used in this study. Two peaks are observed in E01-sfGFP and fusion protein E01-GS-TPD-sfGFP, compared to one single peak in TPD-sfGFP and the negative control DARPin Off7. (**f**) correlation plots for co-cultures stained with E01-sfGFP or fusion protein E01-GS-TPD-sfGFP, in the presence or absence of the anti-EGFR DARPin E69 fused to monomeric Cherry (mCherry). The x-axis corresponds to GFP fluorescence intensity, while the y-axis shows mCherry fluorescence intensity.

Fluorescently tagged versions of the anti-EGFR DARPin E01, the ADAM17 prodomain TPD and the fusion protein E01-GS-TPD were used to determine their binding ability to their respective targets expressed on EGFR-positive/ADAM17-positive A549 cells. Binding was compared to the non-binding maltose binding protein (MBP)-specific DARPin Off7 used as a negative control (Figure 2a). The recombinant proteins bound to intact A549 cells at varying concentrations (Figure 2b). For the fusion protein E01-GS-TPD, the high affinity of E01 to EGFR in contrast to the lower affinity of TPD for ADAM17 resulted in a fusion construct with intermediate affinity. A549 cells made (−/−) for EGFR via CRISPR editing [19] were used to unmask the binding of TPD to ADAM17 at lower concentrations, otherwise cloaked by the higher affinity of the E01 module for EGFR (Figure 2c). Additionally, receptor binding competition assays were performed using fluorescently labeled fusion protein E01-GS-TPD and unlabeled DARPin E01 or TPD at equimolar concentrations. These experiments revealed competition of E01 with E01-GS-TPD for EGFR in A549 EGFR (+/+) cells (Figure 2d). In contrast, competition for EGFR was lost in the A549 EGFR (−/−) cell line where only the TPD monomer competed for ADAM17 binding with the E01-GS-TPD fusion protein. Given the large differences in affinity observed for E01 binding to EGFR compared to TPD binding to ADAM17, we hypothesized that the asymmetric affinities of the modules of our fusion protein to each target could be useful in the targeting of EGFR overexpressing cells that depend on ADAM17 for autocrine signaling, while potentially sparing ADAM17 activity in other cells where EGFR is low or absent. When we examined the distribution of our fusion protein using co-cultures of A549 EGFR (+/+) and EGFR (−/−) cells, we observed that both E01-sfGFP and fusion protein E01-GS-TPD-sfGFP were capable of discriminating between the two populations, while only one peak was observed using TPD-sfGFP (Figure 2e). Co-cultures were also stained with the anti-EGFR DARPin E69-monomeric Cherry (mCherry), a DARPin that binds to an epitope different to E01 [17]; this allows simultaneous binding of E69 and E01 and enables discrimination between EGFR-positive and EGFR-negative cells. A positive correlation was observed in the signal of E69-mCherry and E01-sfGFP, as well as E69-mCherry and fusion protein E01-GS-TPD-sfGFP, indicating enrichment of the fusion protein in the EGFR-positive cells compared to EGFR-negative cells at the tested concentration of 1 µM (Figure 2f). 

### 2.3. Fusion Protein E01-GS-TPD Inhibits EGFR/ADAM17 Activity in Cancer Cells 

**Figure 3:**
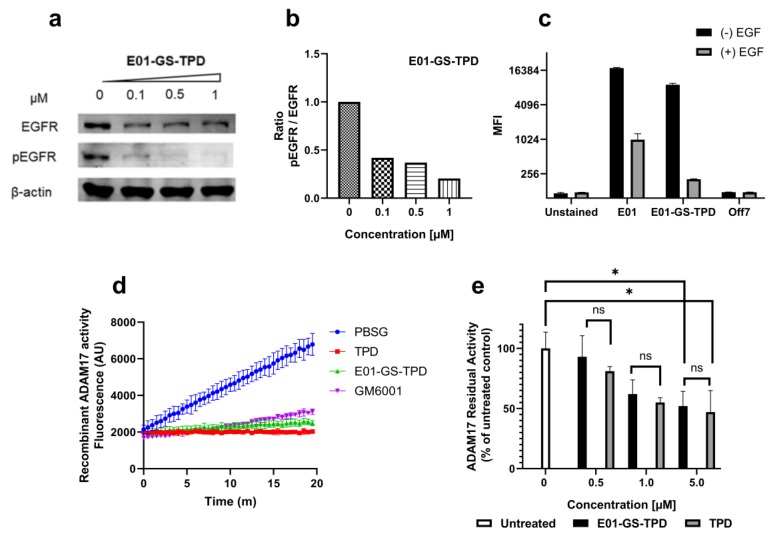
E01-GS-TPD inhibits EGFR phosphorylation and ADAM17 activity in vitro. (**a**) A431 cells were treated with fusion protein E01-GS-TPD at increasing concentrations for a total of 48 h; the levels of total EGFR and phosphorylated EGFR (pTyr1068) were analyzed by Western blot. (**b**) the levels of phosphorylated EGFR relative to total EGFR were calculated based on band intensity and normalized to the untreated control (0 µM)**.** (**c**) to evaluate ligand competition for receptor binding, A431 cells were treated with E01-sfGFP or E01-GS-TPD-sfGFP at 1 µM in the presence or absence of EGF (2 µM). Mean fluorescence intensity was measured. (**d**) to examine the ability of E01-GS-TPD to inhibit ADAM17 activity, recombinant ADAM17 was co-incubated with TPD, E01-GS-TPD, or the broad-spectrum matrix metalloproteinase (MMP) inhibitor GM6001 (2 µM). The increase in fluorescence was monitored, demonstrating inhibition compared to buffer-treated samples. (**e**) additionally, an assay measuring residual activity in live cells using a tumor necrosis factor (TNF)α-based fluorogenic substrate was performed as previously described [20]. E01-GS-TPD displayed a similar ADAM17 inhibitory profile compared to the TPD monomer at the tested concentrations (mean and standard deviation from *n* = 3 where possible is shown, *p < 0.05 in Tukey’s multiple comparisons test).

To validate the capacity of the fusion protein E01-GS-TPD to interfere with the EGFR/ADAM17 axis we used the EGFR-overexpressing cell line A431. Treatment of A431 cells with E01-GS-TPD resulted in a dose-dependent inhibition of basal EGFR phosphorylation, with 1 μM almost completely preventing phosphorylation of EGFR (Figure 3a,b; Appendix C). The inhibitory activity of TPD was retained in the fusion protein E01-GS-TPD as observed in a biochemical assay using human recombinant ADAM17 catalytic domain (Figure 3d). When we measured basal ADAM17 activity, we observed a dose-dependent reduction and a slight decrease of the activity in cells treated with E01-GS-TPD when compared to monomeric recombinant TPD (Figure 3c). These data suggest that our fusion protein achieves inhibition of the EGFR/ADAM17 axis in cancer cells that overexpress EGFR.

### 2.4. Fusion Protein E01-GS-TPD Reduces Pro-Tumorigenic Functions

**Figure 4:**
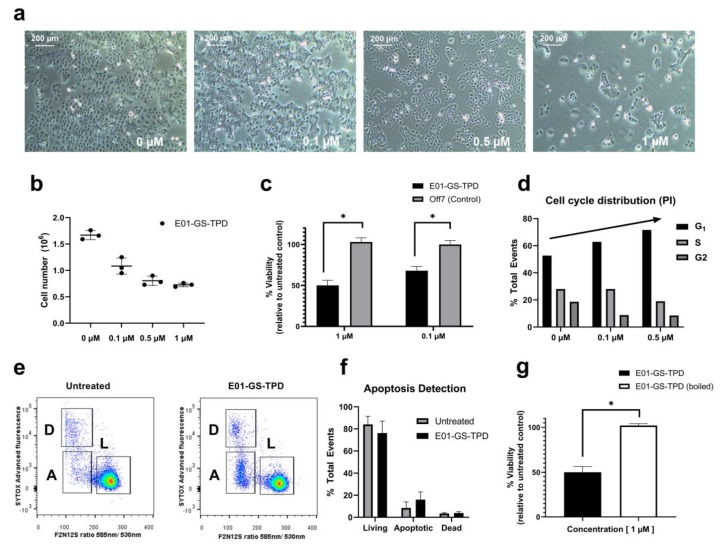
E01-GS-TPD inhibits EGFR/ADAM17-dependent A431 cell proliferation. A431 cells were treated with fusion protein E01-GS-TPD at increasing concentrations for a total of 48 h. (**a**) confluency and (**b)** cell number were examined. (**c**) cell viability following treatment was determined by 3-(4,5-dimethylthiazol-2-yl)-5-(3-carboxymethoxyphenyl)-2-(4-sulphophenyl)-2H-tetrazolium (MTS). (**d**) cell cycle distribution was analyzed using propidium iodide. (**e,f**) apoptosis was detected based on membrane asymmetry to distinguish between dead (D), living (L), and apoptotic cells (A). Mean and standard deviation from (*n* = 3) when shown, * *p* < 0.05 in Tukey’s multiple comparisons test. (**g**) cell viability comparing boiled and non-boiled E01-GS-TPD was determined by MTS.

Treating A431 cells with E01-GS-TPD, we observed a reduced cell density with no apparent increase in the number of dead or floating cells, suggesting decreased proliferation (Figure 4a). Both cell counts and a 3-(4,5-dimethylthiazol-2-yl)-5-(3-carboxymethoxyphenyl)-2-(4-sulphophenyl)-2H-tetrazolium (MTS)-based colorimetric assay confirmed decreased proliferation of A431 cells treated with E01-GS-TPD cells compared to non-binder DARPin Off7 (Figure 4b,c). To evaluate the cause of reduced cell numbers, we measured both cell cycle and apoptosis rates. Cell cycle analysis using propidium iodide staining revealed a dose-dependent increase of cells arrested in the G1 phase, coupled with a decrease of cells found in the S phase (Figure 4d). No significant differences were observed in the percentage of apoptotic cells following E01-GS-TPD treatment, as determined by membrane asymmetry using a ratiometric membrane asymmetry apoptosis detection kit (Figure 4e,f). Finally, to demonstrate dependence of cell growth inhibition on treatment with the native fusion protein, we boiled the fusion protein for 1 h prior to cell treatment. A complete loss of cell growth inhibition was observed in cells treated with boiled E01-GS-TPD, compared to non-boiled E01-GS-TPD (Figure 4g). Put together, these findings suggest E01-GS-TPD mainly decreases the proliferation of viable A431 cells through the inhibition of the EGFR/ADAM17 axis. 

**Figure 5:**
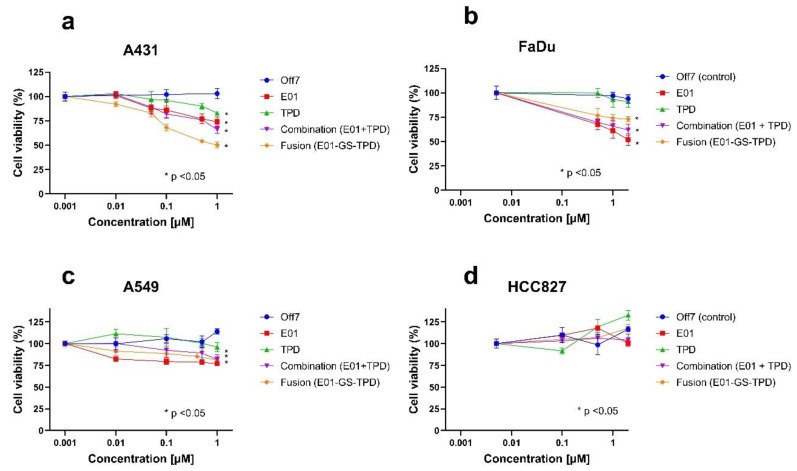
EGFR/ADAM17 inhibition decreases cell proliferation of wild-type EGFR cells. Cell lines were treated with recombinant proteins at the indicated concentrations and allowed to proliferate for 72 h. The largest effects in growth inhibition were observed in the highly overexpressing wild-type EGFR A431 cells (**a**) followed by the wild-type EGFR-positive cell line FaDu (**b**) and the wild-type EGFR, KRAS mutant A549 cell line (**c**). No effects in growth inhibition were observed in the constitutively active EGFR mutant cell line HCC827 (**d**). Mean and standard deviation from *n* = 3 is shown, significance (* *p* < 0.05) was calculated using Tukey’s multiple comparisons test and is shown where applicable for the highest concentration of recombinant protein compared to untreated controls.

To further investigate the anti-tumoral effects of E01-GS-TPD on cell proliferation, additional assays were performed using E01, TPD, fusion protein E01-GS-TPD, or the combination treatment of E01 and TPD in equimolar concentrations. The recombinant proteins were tested at increasing concentrations on epidermoid carcinoma A431 cells, confirming inhibitory roles in cell proliferation (Figure 5a), whereas no effect on growth inhibition was observed for control DARPin Off7 up to 1 µM compared to untreated controls. Furthermore, no significant differences were observed between monomer E01 and E01-GS-TPD (*p*-values > 0.05) at the highest concentration tested. These effects were further confirmed in the EGFR (+/+) squamous cell carcinoma cell line FaDu (Figure 5b), and in the EGFR (+/+)/KRAS mutant cell line A549 (Figure 5c). In contrast, no growth inhibition was observed following treatment of the constitutively active EGFR mutant cell line HCC827 (Figure 5d). Finally, because EGFR/ADAM17 signaling is also implicated in cell migration, we evaluated the effect of E01-GS-TPD on wound healing properties. Inhibition of EGFR/ADAM17 by E01-GS-TPD reduced the migration rate of A431 cells compared to untreated controls (Appendix B). Put together, these findings suggest that E01-GS-TPD is able to reduce the proliferation and migration rates of EGFR (+/+) cells with no significant induction of cell death. 

## 3. Discussion

The ability of bispecific proteins to bind two different epitopes with a single molecule provides several advantages including increased specificity against target cells, the introduction of biological activities to a site of interest, such as recruitment of effector cells, and the delivery of an active payload. However, the functionality of both proteins after genetic fusion is not always conserved as the activity of one protein can be hampered by its fusion partner by means of steric hindrance. In this work, dual specificity for each protein partner was retained in a fusion protein consisting of an EGFR-targeting DARPin fused to the inhibitory prodomain of ADAM17 by a long flexible glycine serine linker. Interestingly, a large disparity in affinities was observed between both effector molecules, with DARPin E01 displaying a much higher affinity for EGFR compared to that of TPD for ADAM17. These observations are in agreement with reports of DARPin E01 displaying high affinity to its target EGFR in the low nanomolar range (K_D_ 0.5 nM), as demonstrated in vitro by surface plasmon resonance (SPR) [16]. In contrast, the affinity of TPD for ADAM17 is largely uncharacterized, presumably due to difficulties of determining protein-protein interactions using an active proteinase. However, previous reports described an inhibitory activity for TPD against recombinant ADAM17 in the nanomolar range (IC_50_ 145 nM), with a drop to low micromolar activity in whole live cells (1–5 µM) [13,21]. As ADAM17 is ubiquitously expressed, a higher affinity towards EGFR overexpressing cells may facilitate targeting of dysregulated autocrine signaling as opposed to inhibition of ADAM17 basal activity. Using co-cultures of EGFR (+/+) and EGFR (−/−) cells, we analyzed the distribution of the fusion protein E01-GS-TPD within mixed populations. E01-GS-TPD successfully discriminated EGFR-positive from EGFR (−/−) cells displaying a similar distribution pattern as monomeric DARPin E01. 

Treatment of the epidermoid carcinoma cell line A431, widely known for its dramatic overexpression of wild-type EGFR [22], resulted in decreased cellular proliferation and migration. Treatment of additional EGFR (+/+) cell lines resulted in decreased cell proliferation, but no significant differences were observed between the fusion protein and its monomer E01, perhaps due to additional proteases involved in EGFR-ligand shedding that were not addressed in this study [6]. As expected, the efficacy of the fusion protein E01-GS-TPD decreased in the KRAS mutant cell lines A549 (Figure 5c) and HCT116. The dependence of EGFR downstream molecular players such as KRAS in the responsiveness to EGFR-targeted therapies is widely reported [23], and lies behind the prescription rationale for similarly working drugs in the clinic, such as the anti-EGFR antibody cetuximab, which is only indicated for wild type KRAS cancers [3]. Similarly, no effects in growth inhibition were observed in the non-small cell lung cancer cell line HCC827, which harbors a mutation in EGFR rendering the receptor constitutively active, regardless of ligand binding [2]. 

## 4. Materials and Methods 

### 4.1. Cell Lines and Culture Conditions

Human epidermoid carcinoma cell line A431, human lung adenocarcinoma cell lines A549 and HCC827, and human pharynx squamous cell carcinoma cell line FaDu were obtained from the American Type Culture Collection (ATCC). A549 EGFR knockout cells (−/−) were kindly provided by B. Liu and H.J. Haisma [19]. All cells were cultured in Dulbecco’s modified Eagle’s medium (DMEM; Gibco, Life Technologies, USA) supplemented with 10% (v/v) fetal bovine serum (FBS), 100 units/mL of penicillin, and 100 µg/mL of streptomycin (Gibco, Life Technologies, USA). Cells were incubated at 37 °C in a humidified incubator supplemented with 5% CO_2_ and atmospheric oxygen. 

### 4.2. Cloning, Production, and Purification of the Recombinant Proteins 

The ORF for anti-EGFR DARPins E01 and E69, the anti-MBP DARPin Off7 [24], and the prodomain of ADAM17 (TPD, Asp^23^-Arg^214^) were digested with restriction enzymes *Bam*HI and *Hind*III (Thermo Fisher Scientific, Netherlands) and ligated into the expression vector pAT222 (GenBank accession number AY327137). Additionally, fluorescent versions of each protein were generated by ligating the ORFs into the vectors pQE30_sfGFP or pQE30_mCherry to yield recombinant proteins fused at the C-terminus to superfolder GFP (sfGFP) [25] or monomeric Cherry [26]. The bispecific construct of DARPin E01 fused to TPD was generated as previously described [18]. Briefly, the C-terminal protein TPD was digested with *Bsa*I and *Bgl*II and subsequently ligated into a pQIBI vector containing a flexible glycine serine (G_4_S)_4_ linker. Subsequently, the N-terminal protein E01 was digested with *Bam*HI and *Hind*III and ligated into the pQIBI vector now containing the C-terminal TPD. The resulting bispecific construct (E01-GS-TPD) was then transferred into the expression vector pAT222 using the restriction enzymes *Bam*HI and *Bsa*I. After transformation of *Escherichia coli* BL21(DE3), all proteins were produced and purified via their 6xHis-Tag with nickel-nitrilotriacetic acid superflow resin (Ni-NTA, Qiagen, Hilden, Germany). The eluate was purified further via size exclusion chromatography (Superdex75 16/60), using TBSG buffer (100 mM Tris-HCl, pH 8.2, 300 mM NaCl, and 10% (v/v) glycerol). The purified recombinant protein was immediately frozen in liquid nitrogen and stored at −80 °C until further use.

### 4.3. Western Blot Analysis 

A431 cells were seeded in six-well plates at a density of 0.5 × 10^6^ cells/well. Cells were maintained in DMEM supplemented with 10% (v/v) FBS. After 24 h, the medium was changed to 1% (v/v) FBS for serum starvation and incubated in the presence or absence of recombinant proteins for an additional 24 h. Following treatment, cells were washed and lysis buffer was added (50 mM Tris-HCl, pH 7.4, 150 mM NaCl, and 0.5% (w/v) sodium deoxycholate, 0.1% (v/v) Nonidet P-40, 0.1% (v/v) SDS, 1 mM sodium orthovanadate, and 1 PhosSTOP tablet (Roche Applied Science, Germany)). Total protein concentration for each cell lysate was determined using a bicinchonic acid (BCA) protein assay kit (Pierce, Rockford IL, USA), and 30 μg of total protein per sample was separated in 10% SDS-PAGE and transferred to an Immobilon FL membrane (Millipore). After membrane blocking with Odyssey blocking buffer (LI-COR Biosciences GmbH, Bad Homburg, Germany), the membranes were probed with anti-phospho-EGFR (pTyr1068) (Cell Signaling Technologies Beverly, MA, USA) or anti-EGFR (Santa Cruz Biotechnology, USA) antibodies. β-actin was detected as a protein loading control using an anti-β-actin antibody (Cell Signaling Technologies). The membranes were subsequently incubated with IRDye-conjugated secondary antibodies (800 CW, Goat anti-Mouse; 680RD Goat anti-Rabbit; LI-COR). Protein bands were detected with Odyssey software (LI-COR) and analyzed using ImageJ.

### 4.4. ADAM17 Inhibition Assays Using a Fluorogenic Substrate

To confirm inhibition of ADAM17 activity, purified recombinant ADAM17 was used in the presence or absence of recombinant protein, or the commercial inhibitor GM6001 following manufacturer’s instructions (ADAM17 fluorometric drug discovery kit, Enzo Life Sciences, Inc). To determine inhibition of ADAM17 activity in live cells fluorescence-based assays were performed as previously described [20]. Briefly, A431 cells were seeded in black 96-well plates at a density of 2.5 × 10^4^ cells/well. The cells were allowed to adhere by incubating them at 37 °C for 2 h. Following the incubation period, the culture medium was removed, and cells were treated with the recombinant proteins (0.5–5 μM) at 37 °C for 30 min to allow binding to their cellular target. The TNFα-based quenched fluorogenic substrate FAM–SPLAQAVRSSSRK–TAMRA was added to each well to a final concentration of 5 μM (BACHEM, Weil am Rhein, Germany). Cleavage of the quenched peptide substrate by mature ADAM17 resulted in unquenching and an increase in fluorescence. Fluorescence was measured at 520 nm every minute using a FLUOstar Optima BMG Labtech plate reader. The activity of mature ADAM17 was calculated as the increase of fluorescence units per minute over a 20-min period in the linear phase of the reaction. Last, the inhibitory activity of each protein was calculated as the percentage of residual activity of treated cells relative to untreated cells. 

### 4.5. Cell-Based Target Binding Assays

Analytical flow cytometry experiments were performed to examine the binding of the recombinant proteins to their cellular targets. To this end, wild type A549 (+/+) or EGFR knockout cells (−/−) were trypsinized, washed twice with PBS, and counted using a hemocytometer. Approximately 2.5 × 10^5^ cells per condition were incubated at 37 °C for 30 min with 1 μM fluorescent recombinant protein. Following incubation, cells were washed twice, resuspended in FACS buffer (PBS pH 7.4, 10% (v/v) FBS), and kept on ice until analysis. Fluorescence intensity was examined by flow cytometry using a BD Biosciences LSR-II system. Finally, the generated FACS data were analyzed using Kaluza Analysis flow cytometry software (Beckman Coulter). 

To determine the specificity of the recombinant proteins, competition experiments were performed. Wild type A549 (+/+) or EGFR knockout cells (−/−) were trypsinized, washed twice with PBS, and counted using a hemocytometer. Approximately 2.5 × 10^5^ cells per condition were incubated at 37 °C for 30 min with 2 μM fluorescent recombinant protein and 2 μM of the same recombinant yet unlabeled protein. Following the incubation, cells were washed twice with FACS buffer, and the fluorescence was measured by flow cytometry. To confirm ligand competition for receptor binding ± EGF competition assays were performed. In short, A431 cells were trypsinized, washed twice with PBS, and incubated on ice with or without EGF for 20 min. Cells were next washed twice, fluorescence was measured using a BD FACSVerse ^TM^ and analyzed using Kaluza Analysis flow cytometry software.

### 4.6. Cell Viability Assays 

Cell viability was measured using a colorimetric assay based on conversion of MTS to formazan by metabolically active cells (Promega, Netherlands). Cells were seeded in DMEM (2% (v/v) FBS) into 96-well plates at a density of 10,000 cells per well in triplicates and allowed to adhere overnight. For growth inhibition assays cells were treated with recombinant proteins (0.001–1 μM) and incubated for 72 h to allow proliferation. Absorbance was measured at 490 nm and expressed as a percentage of the untreated controls.

### 4.7. Cell Cycle Analysis and Apoptosis Detection

Cells were seeded onto six-well plates at a density of 0.5 × 10^6^ cells/well in DMEM containing 10% (v/v) FBS. After overnight incubation cells were starved of FBS (down to 2% (v/v)) and treated with recombinant protein for 24 h. Cells were then trypsinized, washed twice, and permeabilized using 70% (v/v) ethanol prior to propidium iodide staining [27]. Cells were next analyzed using a BD Biosciences FACS Calibur. 

For apoptosis detection, the Violet Ratiometric Membrane Asymmetry Probe/Dead cell Apoptosis Kit (Thermo Fisher Scientific, Netherlands) was used as indicated by the manufacturer and apoptosis was analyzed using a BD Biosciences LSR-II. Single cells were gated, and the resulting DNA distributions were analyzed using the flow cytometry analysis software FlowJo. 

## 5. Conclusions

In conclusion, we provide evidence that dual inhibition of EGFR and ADAM17 can be achieved with a single molecule using the fusion protein E01-GS-TPD. E01-GS-TPD was successful in inhibiting the proliferation of EGFR-dependent cell lines with a wild-type KRAS background. As increased ADAM17 activity constitutes a known mechanism of acquired resistance in response to chemotherapy, ionizing radiation, and EGFR inhibition [12,28], future (animal) studies using E01-GS-TPD targeted dual inhibition of EGFR and ADAM17 are required to evaluate its performance compared to mono-targeted therapies. 

## Figures and Tables

**Figure A1:**
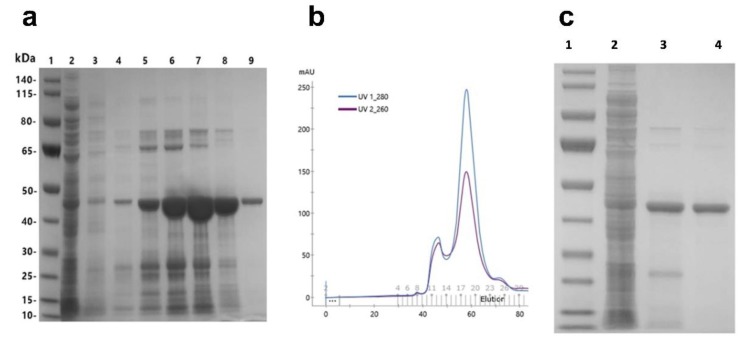
Purification procedure of recombinant E01-GS-inhibitory prodomain of ADAM17 (TPD). (**a**) following protein production in *E. coli* BL21(DE3), bacteria were lysed by sonication. The resulting lysates were clarified by centrifugation and loaded onto Ni-NTA resin for affinity chromatography and analyzed by SDS-PAGE: (**a1**) molecular weight protein ladder, (**a2**) flow through, (**a3**) wash (10 mM imidazole), and (**a4**–**9**) recombinant protein eluates using increasing concentrations of imidazole (50–1000 mM). (**b**) the eluate from affinity chromatography was further purified by size exclusion chromatography (Superdex75 16/60) with the monomeric fusion protein eluting in one sharp single peak. (**c)** the purity of the recombinant protein was assessed via polyacrylamide gel electrophoresis (SDS-PAGE): (**c1**) protein ladder, (**c2**) bacterial cell lysate, (**c3**) eluate affinity chromatography, and (**c4**) eluate size exclusion chromatography.

**Figure A2:**
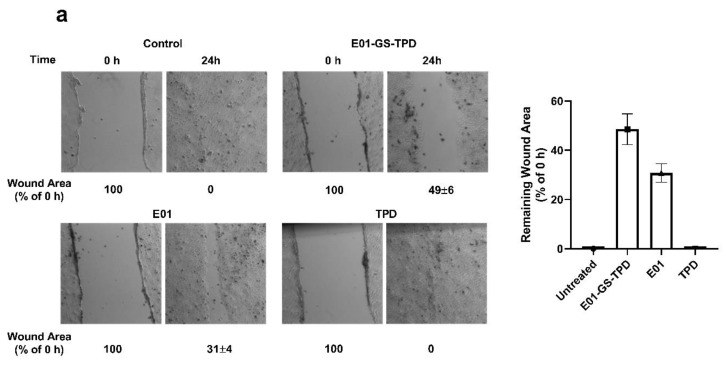
E01-GS-TPD inhibits A431 cell migration. Scratches were made in confluent A431 cultures to create a visible wound in the cell monolayer. Cultures were treated with recombinant protein at a final concentration of 0.5 µM for 24 h and the remaining wound area was compared to the initial wound at 0 h (**a**). The remaining wound area for each condition was then calculated to determine the effects of each protein on cell migration (**b**). Mean and standard deviation from *n* = 2 is shown.

**Figure A3:**
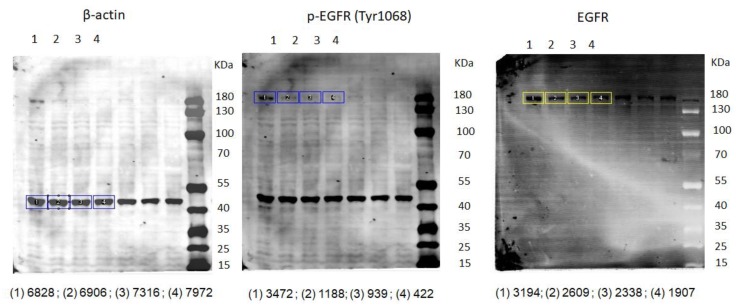
Western blot and densitometry analysis of A431 cells treated with E01-GS-TPD. Western blot pictures from Figure 3a are shown. The densitometry analysis for each sample is shown below each blot (1) untreated 0 µM, (2) E01-GS-TPD 0.1 µM, (3) E01-GS-TPD 0.5 µM, and (4) E01-GS-TPD 1 µM.

## Appendix B

### B.1. Wound Healing Assays

To determine the effects of the recombinant proteins on migration capacity, A431 cells were seeded in six-well plates at a density of 1 × 10^6^ cells/well in Dulbecco’s modified Eagle’s medium (DMEM) containing 10% (v/v) fetal bovine serum (FBS). Cells were allowed to adhere and divide for 24 h to create a confluent monolayer. Cells were then starved of FBS and a wound was created by scraping the cell monolayer in a straight line with a sterile pipette tip. Debris was removed by washing with fresh medium devoid of FBS. The different recombinant proteins were next added to each well and the plates were placed in a tissue culture incubator at 37 °C. Pictures were acquired using a CKX41 inverted microscope (Olympus, Japan) using the Leica Application Suite software (Leica Microsystems Limited, Switzerland). Subsequent pictures were taken following incubation until the scratch of the control group had healed completely after 24 h. The remaining wound area of treated samples at 24 h was compared to the initial wound area prior to treatment at 0 h and expressed as a percentage relative to the start (0 h).
